# ^13^C metabolic flux analysis-guided metabolic engineering of *Escherichia coli* for improved acetol production from glycerol

**DOI:** 10.1186/s13068-019-1372-4

**Published:** 2019-02-13

**Authors:** Ruilian Yao, Jiawei Li, Lei Feng, Xuehong Zhang, Hongbo Hu

**Affiliations:** 1 State Key Laboratory of Microbial Metabolism, and School of Life Sciences and Biotechnology, Shanghai Jiao Tong University, 800 Dongchuan Road, Shanghai, 200240 China; 2 Instrumental Analysis Center, Shanghai Jiao Tong University, 800 Dongchuan Road, Shanghai, 200240 China

**Keywords:** ^13^C metabolic flux analysis, Metabolomics, Acetol, Glycerol, NADPH, *Escherichia coli*

## Abstract

**Background:**

Bioprocessing offers a sustainable and green approach to manufacture various chemicals and materials. Development of bioprocesses requires transforming common producer strains to cell factories. ^13^C metabolic flux analysis (^13^C-MFA) can be applied to identify relevant targets to accomplish the desired phenotype, which has become one of the major tools to support systems metabolic engineering. In this research, we applied ^13^C-MFA to identify bottlenecks in the bioconversion of glycerol into acetol by *Escherichia coli*. Valorization of glycerol, the main by-product of biodiesel, has contributed to the viability of biofuel economy.

**Results:**

We performed ^13^C-MFA and measured intracellular pyridine nucleotide pools in a first-generation acetol producer strain (HJ06) and a non-producer strain (HJ06C), and identified that engineering the NADPH regeneration is a promising target. Based on this finding, we overexpressed *nadK* encoding NAD kinase or *pntAB* encoding membrane-bound transhydrogenase either individually or in combination with HJ06, obtaining HJ06N, HJ06P and HJ06PN. The step-wise approach resulted in increasing the acetol titer from 0.91 g/L (HJ06) to 2.81 g/L (HJ06PN). To systematically characterize and the effect of mutation(s) on the metabolism, we also examined the metabolomics and transcriptional levels of key genes in four strains. The pool sizes of NADPH, NADP^+^ and the NADPH/NADP^+^ ratio were progressively increased from HJ06 to HJ06PN, demonstrating that the sufficient NADPH supply is critical for acetol production. Flux distribution was optimized towards acetol formation from HJ06 to HJ06PN: (1) The carbon partitioning at the DHAP node directed gradually more carbon from the lower glycolytic pathway through the acetol biosynthetic pathway; (2) The transhydrogenation flux was constantly increased. In addition, ^13^C-MFA showed the rigidity of upper glycolytic pathway, PP pathway and the TCA cycle to support growth. The flux patterns were supported by most metabolomics data and gene expression profiles.

**Conclusions:**

This research demonstrated how ^13^C-MFA can be applied to drive the cycles of design, build, test and learn implementation for strain development. This succeeding engineering strategy can also be applicable for rational design of other microbial cell factories.

**Electronic supplementary material:**

The online version of this article (10.1186/s13068-019-1372-4) contains supplementary material, which is available to authorized users.

## Background

The shortage of non-renewable energy sources and their negative impacts on the environment have inspired interests in the field of bio-based economy [1]. The use of microbial systems for manufacturing different kinds of chemicals offers a sustainable and promising approach [2]. To transform commercial producer strains to cell factories, systems metabolic engineering is required to maximize the overall production yield, titer, and productivity [3, 4].

Systems biology aims at acquiring a comprehensive and holistic understanding of cellular mechanisms by employing multi-omics data and computational simulations [3, 5]. It plays important roles in the design and optimization of the cell factories and bioprocess for years [6, 7]. Recently, fluxomics, reflecting the integrated outputs of gene–protein–metabolite interactions [8], has attracted significant attentions since the ultimate purpose of metabolic engineering is to reroute flux towards chemical production [4]. ^13^C-MFA can accurately determine the flux configuration with the help of isotope tracers, providing information on relative contributions of different metabolic routes, in particular, for the generation of energy and cofactors [9–12]. ^13^C-MFA has been effectively applied to identify relevant targets to accomplish the desired phenotype [11, 13–16]. Jazmin et al. [13] used isotopically nonstationary ^13^C metabolic flux analysis to identify the pyruvate kinase (PK) and enzymes in the PK bypass pathway as overexpression targets to debottleneck isobutyraldehyde production in cyanobacteria. Okahashi et al. [11] performed ^13^C-MFA in isopropyl alcohol (IPA)-producing *E. coli* strains and identified NADPH regeneration as a target. To debottleneck IPA production, the authors successfully engineered *E. coli* strains under nitrogen-starved culture conditions that produced 41.6 mM IPA at a yield of 0.55 (mol/mol) from glucose.

Acetol is mainly used as an important organic intermediate to produce polyols and acrolein [17]. It is also utilized as a reducing agent in the textile industry [18]. Chemical and biological approaches have been explored to produce acetol [19–22]. However, chemical processes are petroleum based. Bioconversion of renewable feedstocks into acetol is more competitive owing to its economically attractive and environmentally friendly properties. Soucaille et al. [23] reported the development of a metabolic engineered *E. coli* strain that produced 1.63 g/L of acetol from 9.6 g/L glucose in the minimal medium. Valorization of glycerol, the waste stream of biodiesel production, has contributed to the viability of biofuel economy [24, 25]. The metabolic pathways for acetol biosynthesis in *E. coli* using glycerol as the carbon source are shown in Additional file 1. Acetol is formed from DHAP in the two reaction steps catalyzed by methylglyoxal synthase (MgsA) and NADPH-dependent aldehyde oxidoreductase (YqhD).

In our previous work, we facilitated the biosynthesis of acetol in the HJ02 strain derived from *E. coli* BW25113 by replacing the native *glpK* gene with the allele from *E. coli* Lin43 and overexpressing *yqhD* [22]. In addition, derivatives of HJ02 strain were constructed and the final strain produced 1.82 g/L of acetol. However, the titer was suboptimal and improvable. In this study, we performed ^13^C-MFA and measured intracellular pyridine nucleotide pools in a first-generation acetol producer strain and a non-producer strain to interrogate mechanisms behind acetol production, and to identify possible targets for genetic modifications. The results revealed that engineering the NADPH regeneration is a promising target. Based on this finding, we employed cofactor engineering to increase acetol production. To systematically characterize and understand the effect of mutation(s) on the metabolism, we also examined the metabolomics and transcriptional levels of key genes in optimized strains and the parental strain.

## Results

### ^13^C metabolic flux analysis in first-generation acetol producer and non-producer strains

**Fig. 1:**
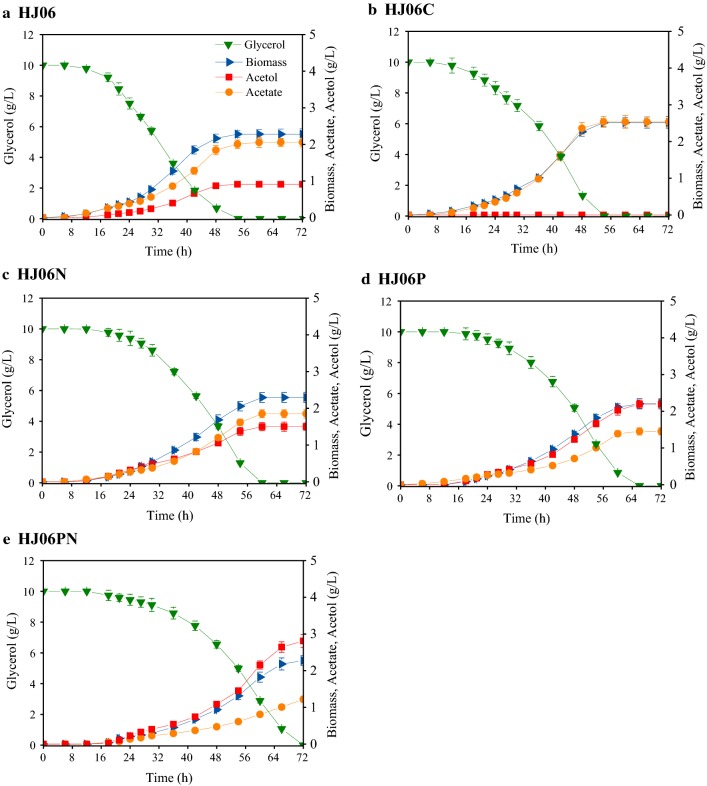
Culture profiles of acetol-producing and non-producing *E. coli* strains HJ06 (**a**), HJ06C (**b**), HJ06N (**c**), HJ06P (**d)**, and HJ06PN (**e**). Data represent the mean ± SD from three independent cultures

**Fig. 2:**
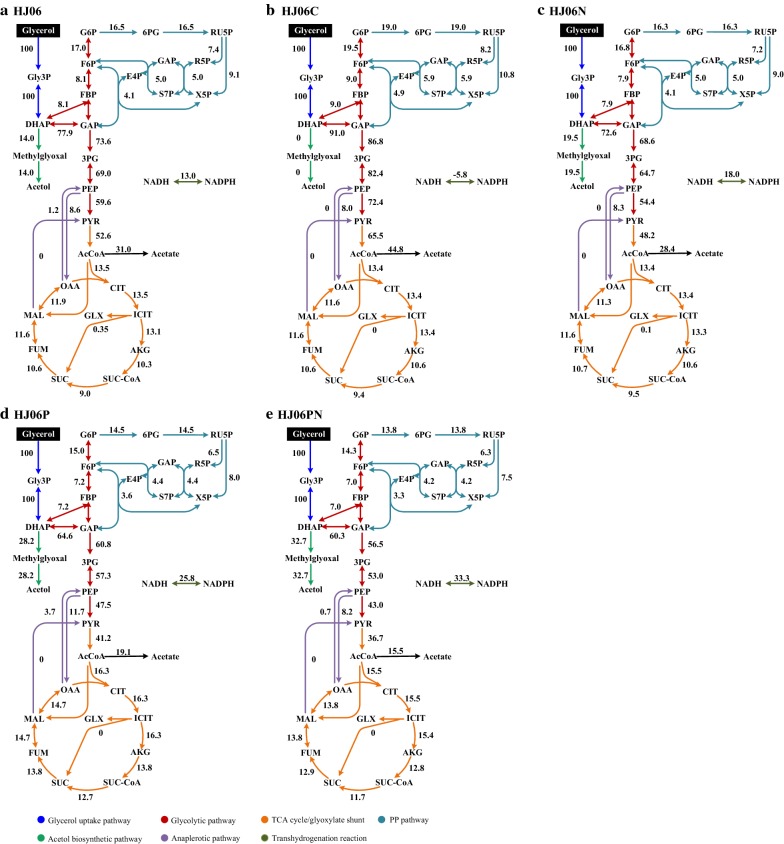
Flux distributions in acetol-producing and non-producing *E. coli* strains HJ06 (**a**), HJ06C (**b**), HJ06N (**c**), HJ06P (**d)**, and HJ06PN (**e**). The flux values of the best fit were normalized to the specific glycerol consumption rate of 100

An acetol-producing strain (HJ06) was constructed by silencing of *gapA* from HJ02 [22], which produced 0.91 g/L of acetol from glycerol (Fig. 1a). HJ06 was used as a platform for the optimization of acetol production. Its control strain (HJ06C) was constructed by replacing the *glpK*, silencing of *gapA*, and knocking out of *mgsA* in *E. coli* BW25113, which did not produce acetol (Fig. 1b). We analyzed both our first-generation strain, HJ06, and the control strain, HJ06C, using ^13^C-MFA to identify bottlenecks for the acetol production. It was reported that [1,3-^13^C]glycerol can resolve key fluxes with high precision in *E. coli* [26]. Thus, we chose this tracer as a substrate. The best-fit flux maps are shown in Fig. 2. The experimentally measured and model-simulated intracellular metabolite MIDs, and confidence intervals are listed in Additional files 2 and 3. All ^13^C-MFA performed in this study passed *χ*^2^ tests (Additional file 4). In both strains, the relative flux through the pentose phosphate (PP) pathway was low and acetate overflow exceeded the TCA cycle flux (Fig. 2a, b). We identified the flexibility of the DHAP node where flux was re-routed to accommodate increased acetol formation. Compared with HJ06C, the flux through triose-phosphate isomerase was decreased by 14.4% in HJ06. The reversal of the transhydrogenation flux (NADPH → NADH in HJ06C versus NADH → NADPH in HJ06) pointed towards the shortage of NADPH supply from other pathways in HJ06.

**Fig. 3:**
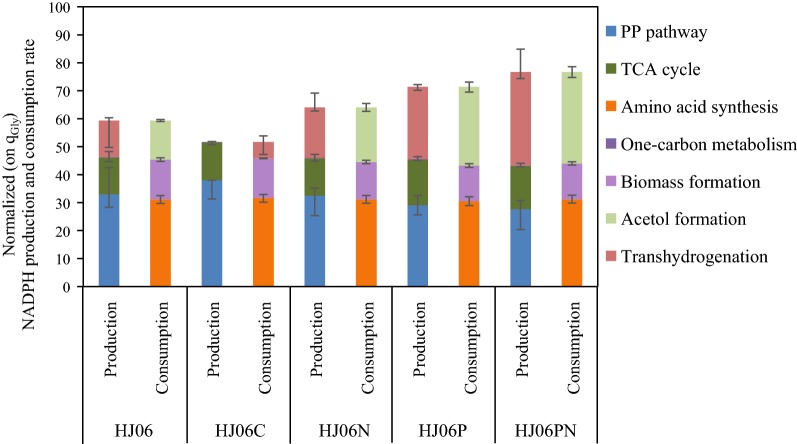
Estimated production and consumption of NADPH by acetol-producing and non-producing *E. coli* strains. *q*_Gly_, the specific glycerol consumption rate

Metabolism-wide NADPH production and consumption in HJ06 and HJ06C were then estimated based on metabolic fluxes (Fig. 3). The major NADPH-generating reactions are glucose-6-phosphate dehydrogenase and 6-phosphogluconate dehydrogenase in the oxidative PP pathway, isocitrate dehydrogenase in the TCA cycle, and transhydrogenase (PntAB and UdhA) [27]. Since the NADPH was only required for anabolic demand in HJ06C, excess NADPH was converted to NADH by the transhydrogenase flux. In HJ06, the fluxes through the PP pathway and the TCA cycle produced less NADPH than was required for biomass and acetol biosynthesis. There was a 21.9% gap to be filled to fulfill the demand for NADPH.

**Fig. 4:**
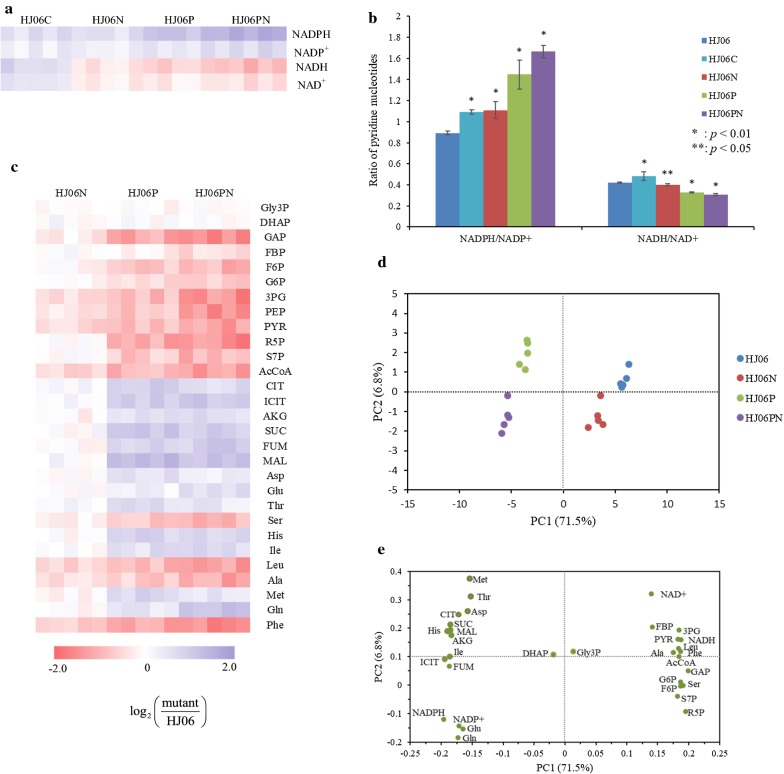
Aggregate metabolomics data and representative analyses. **a** Heat map of pyridine nucleotide pools in acetol-producing and non-producing *E. coli* strains (*n* = 5). **b** Specific changes in NAD(P) metabolite ratios. **c** Heat map of metabolite levels in acetol-producing *E. coli* strains (*n* = 5). **d** PCA score plot for metabolic profiling of acetol-producing and non-producing *E. coli* strains. **e** The corresponding loading plot illustrating metabolites that contributed to the separation on PC1 and PC2. *P* values were obtained using a *t* test comparing optimized strains with the HJ06

The aforementioned results indicated that the supply of NADPH was a bottleneck for the acetol overproduction. To confirm this hypothesis, we measured intracellular pyridine nucleotide pools in both strains and calculated the NADPH/NADP^+^ ratio (Fig. 4a, b; Additional file 5). Compared with HJ06, the NADPH and NADP^+^ concentrations, and the NADPH/NADP^+^ ratio were higher in HJ06C. Taken together, these data allowed us to identify that engineering the NADPH regeneration is a promising target for the acetol overproduction.

### Overexpressing *nadK* to improve acetol production

In *E. coli*, ATP-dependent NAD kinase encoded by *nadK* converts NAD^+^ to NADP^+^ [28]. Overexpression of *nadK* increased NADPH supply, which is beneficial for NADPH-dependent biosynthetic pathways, such as thymidine in *E. coli* [29], l-arginine in *C. crenatum* [30], and l-isoleucine in *C. glutamicum* [31]. In this work, the effect of overexpressing *nadK* on acetol production was investigated by introducing the expressing vector pBbB5K-*nadK* into HJ06. The resulting strain was named HJ06N. HJ06N had a 6-h longer delay for growing in the mineral media than the base strain, HJ06. Once adapted, the specific growth rate and the specific glycerol consumption rate were comparable in HJ06 and HJ06N (Table 1). HJ06N produced 1.50 g/L of acetol (Fig. 1c), a 65% higher value as compared to HJ06. This strain showed slight decreases (~ 10%) in both acetate concentration and the specific acetate production rate when compared to HJ06 (Fig. 1c, Table 1).

**Table 1:** Growth kinetic parameters of acetol-producing and non-producing *E. coli* strains

Strain	Specific growth rate (h^−1^)	Specific rate (mmol/g/h)			*Y*_A/G_ (mol/mol)
		Glycerol consumption	Acetol production	Acetate production	
HJ06	0.07 ± 0.002	2.75 ± 0.06	0.38 ± 0.01	0.86 ± 0.02	0.11 ± 0.003
HJ06C	0.07 ± 0.001	2.81 ± 0.06	0	1.30 ± 0.03	0
HJ06N	0.07 ± 0.002	2.78 ± 0.07	0.54 ± 0.01	0.79 ± 0.02	0.19 ± 0.003
HJ06P	0.06 ± 0.001	2.61 ± 0.06	0.74 ± 0.02	0.50 ± 0.01	0.27 ± 0.004
HJ06PN	0.06 ± 0.001	2.58 ± 0.06	0.86 ± 0.02	0.40 ± 0.01	0.35 ± 0.005

*Y*_A/G_, acetol yield on glycerol

To study the relationship between the NADPH regeneration and the activation of acetol production in HJ06N, a metabolic flux distribution was determined by ^13^C-MFA (Fig. 2c). The strain HJ06N exhibited a slight higher flux shift from the lower glycolytic pathway to the acetol biosynthetic pathway. The flux through the PP pathway and the TCA cycle was very similar in HJ06 and HJ06N. The transhydrogenation flux converting NADH to NADPH increased 1.4-fold compared to HJ06 (Figs. 2c and 3).

### Overexpressing *pntAB* to improve acetol production

The membrane-bound transhydrogenase PntAB transfers a hydride from NADH to NADP^+^ with the concurrent production of NADPH and NAD^+^ in an energy-dependent manner [27]. Overexpressing *pntAB* has been used to improve NADPH availability for improving NADPH-derived products, such as shikimate [32] and poly-(3-hydroxybutyrate) [33]. We hypothesized that overexpressing *pntAB* in HJ06 would increase the transhydrogenase flux and subsequently increase acetol production. Thus, we generated the *pntAB*-overexpressing strain called HJ06P. The specific growth rate and the specific glycerol consumption rate were not substantially affected in HJ06P (Table 1). The acetol production in HJ06P was increased to 2.20 g/L, 2.4 times higher than that in HJ06 (Fig. 1d). The amount of acetate formed was reduced about 29% when compared to HJ06.

^13^C-MFA revealed that the strain HJ06P channeled significantly more carbon flux from lower glycolysis to the acetol biosynthetic pathway (Fig. 2d). Compared with HJ06, the fluxes through the PP pathway and the TCA cycle were slightly changed in HJ06P. The transhydrogenase flux was further increased, accounting for 36.2% of the NADPH supply (Figs. 2d and 3).

### Co-overexpressing *nadK* and *pntAB* to further improve acetol production

Considering the increased production in the single-gene overexpression strains, we hypothesized that combinatorial overexpression of *pntAB* and *nadK* could have a synergetic effect. To test this hypothesis, the HJ06PN strain harboring the recombinant plasmid pBbB5K-*pntAB*-*nadK* carrying the expression cassettes for *pntAB* and *nadK* was cultivated to study the effect of overexpression of these genes on acetol production. HJ06PN did not substantially modify the specific growth rate and the specific glycerol consumption rate (Table 1). The best producing strain HJ06PN accumulated up to 2.81 g/L of acetol (Fig. 1e), which was 3.1 times of that in the original strain, HJ06. Concomitantly, the resulting yield of acetol on glycerol (*Y*_A/G_) and the specific acetol production rate in HJ06PN were superior to other three strains (Table 1). It is also important to emphasize that the lowest acetate production and rate occurred in HJ06PN, which were 41% and 53% lower than the values obtained from HJ06 (Fig. 1e, Table 1).

^13^C-MFA was performed to determine the flux distribution in strain HJ06PN (Fig. 2e). In HJ06PN, the distributions of key fluxes through the PP and TCA cycle were almost identical to HJ06P. The transhydrogenase reaction became a major pathway of NADPH formation that contributed to 43.5% of the total demand for NADPH (Figs. 2e and 3).

### Metabolome analysis of acetol-producing strains

To further evaluate the systematic alterations that contribute to acetol production, we performed isotope-assisted LC–MS-based metabolome analysis in all strains. Four sets of the metabolomics profiles each with 33 key metabolites were detected and the datasets were subjected to principle component analysis (PCA) (Fig. 4, Additional file 5). Strains were clearly separated based on their *pntAB* genotype in the first component and also on their *nadK* genotype in the second component (Fig. 4d). HJ06N displayed milder metabolic changes, and HJ0P and HJ06PN displayed strong metabolic changes. The most striking and global change in the metabolic state was induced by the combinatorial overexpression strategy. Then, metabolites contributing to the separation of samples were examined in the loading plot (Fig. 4e). Separation between strains was greater along PC1 (71.5%), where NADH, NAD^+^, metabolites in the glycolytic pathway and PP pathway, and amino acids synthesized from these precursors showed positive loadings, NADPH, NADP^+^, and metabolites involved in the TCA cycle, and their derived amino acids showed negative loadings. PC2 explained 6.8% of the variability, where metabolites with higher contributions to this component were Met, NAD^+^ and Thr in the positive direction.

A *t* test was performed comparing optimized strains and the first-generation producer strain, aiming to identify which metabolites have significant differences. Overexpression of *nadK* and *pntAB* had a profound influence on the cellular redox status as expected (Fig. 4; Additional file 5). The NADPH and NADP^+^ concentration as well as the NADPH/NADP^+^ ratio was most significantly elevated in HJ06PN, while NADH and NAD^+^ levels as well as the NADH/NAD^+^ ratio showed an opposite trend in this strain. The changes in the metabolites in the central carbon metabolism and amino acids were only moderate (up to 2.6-fold). Gly3P and DHAP in the glycerol uptake pathway did not show differences between all strains. The levels of upper glycolytic pathway intermediates (F6P and G6P) and PP pathway intermediates (R5P and S7P) were not significantly altered in HJ06N when compared with HJ06, but their levels were lower in HJ06P and HJ06PN than in HJ06. The FBP pool was similar in HJ06, HJ06N and HJ06P, but was present in lower amount in HJ06PN. The upper glycolytic pathway intermediate GAP and the lower glycolytic pathway intermediates (3PG, PEP and PYR) and the TCA cycle metabolite (AcCoA) were less abundant in the optimized strains than in HJ06. Other TCA cycle metabolites, including CIT, ICIT, AKG, SUC, FUM, MAL in HJ06N, were not significantly different from that in HJ06, but were present in higher concentrations in HJ06P and HJ06PN than in HJ06. The changing patterns of amino acids might be associated with their precursors.

### Transcriptional analysis of acetol-producing strains

**Fig. 5:**
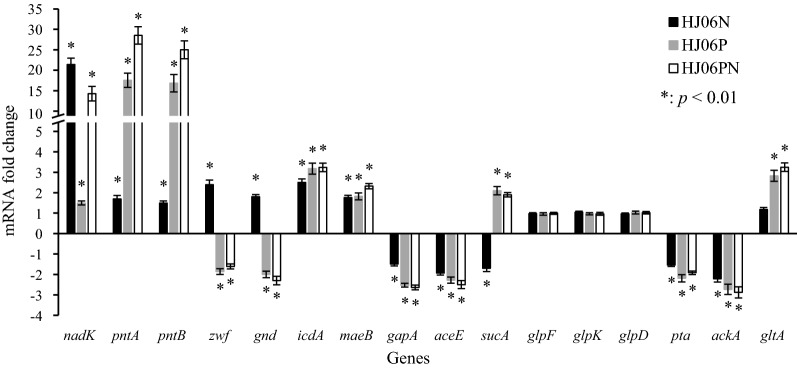
Fold changes of transcription levels of selected genes in optimized strains compared with HJ06. Data represent the mean ± SD from five independent cultures

To gain a deeper insight of metabolic changes caused by redox equivalent, we measured the transcriptional levels of selected genes in optimized strains by qRT-PCR and normalized the data against the values measured from HJ06 (Fig. 5). Expression analysis also demonstrates that the above modifications indeed enhanced the transcription levels of the *nadK* and *pntAB* genes. Compared with HJ06, the expression levels of genes encoding NADP^+^-dependent dehydrogenases, *zwf*, *gnd*, *icdA*, and *maeB*, and *pntA* were upregulated in HJ06N, thereby reducing excess NADP^+^ to overproduce NADPH. In contrast, genes encoding NAD^+^-dependent dehydrogenases, *gapA*, *aceE*, and *sucA,* were transcriptionally attenuated in HJ06N. In addition, the transcript levels of the glycerol uptake pathway genes, *glpF*, *glpK*, and *glpD,* were not significantly altered, and the acetate production pathway genes, *pta* and *ackA,* were downregulated in HJ06N, which correlate to the constant glycerol consumption rate and decreased acetate production rate. Moreover, the expression level of *gltA* in the TCA cycle was not modified in HJ06N, consistent with the flux distribution. In contrast to the HJ06N/HJ06 comparison, *zwf* and *gnd* were found to be downregulated, and *sucA* and *gltA* were found to be upregulated during the HJ06P/HJ06 and HJ06PN/HJ06 comparisons, which agreed with changes of fluxes. Expression profiles between HJ06N/HJ06, HJ06P/HJ06 and HJ06PN/HJ06 showed no significant differences in the regulation of other genes.

## Discussion

### Cofactor engineering and metabolism

Systems biology has proven valuable to uncover the target selection in metabolic engineering [34]. The present study is a good example of using ^13^C-MFA to guide rational engineering for strain development. ^13^C-MFA together with analysis of redox states revealed that NADPH regeneration is a limiting step, and the NADPH availability and well redox balance status are crucial for efficient acetol biosynthesis. We tested our hypothesis by overexpressing *nadK* and *pntAB* either individually or in combination in HJ06, an engineered strain in our previous work [22]. The step-wise approach resulted in increasing the acetol titer from 0.91 to 2.81 g/L and the transhydrogenation flux from 13.0 to 33.3%. These findings suggested that the supply of NADPH for acetol production was clearly improved by our strategies.

In addition to the direct genetic modifications for maximizing target metabolite pathway flux, cofactor engineering is an effective tool to improve redox status and finally promoted the product biosynthesis [29, 32, 33, 35]. Several groups tried to increase NADPH supply through the engineering of the PP pathway [36–38]. However, the PP pathway releases one carbon as CO_2_, leading to the decreased production yield. In addition, the enhancement of the PP pathway could influence the flux of the central metabolic pathway as well as the production of certain compounds. Overexpressing NAD^+^ kinase or transhydrogenase is an alternative way to improve NADPH supply, in turn increasing the production of thymidine [29], l-arginine [30], l-isoleucine [31], shikimate [32], and poly-(3-hydroxybutyrate) [33]. In our approach, the strain with *pntAB* overexpression showed better performance than *nadK* overexpression, indicating that PntAB is more advantageous to enhance acetol production compared with NAD kinase. In addition, the double gene overexpression strain (HJ06PN) had higher acetol production than the best single-gene overexpression strain (HJ06P). The strategy of co-overexpression of *pntAB* and *nadK* was also useful for improving the production of isobutanol and fatty acid [35, 39].

The intracellular levels of redox equivalents were measured to evaluate the effect of cellular status on the acetol biosynthesis. NAD kinase represents itself a key enzyme for controlling NAD(H) and NADP(H) balance [40]. PntAB generates NAD^+^, which can be phosphorylated to NADP^+^ by NAD kinase. Thus, combining NAD^+^ kinase and PntAB can push the reaction direction towards NADPH biosynthesis. The pool sizes of NADPH, NADP^+^ and the NADPH/NADP^+^ ratio were progressively increased from first-generation producer strain to final highest-producer strain, accompanied by the enhanced transhydrogenase flux, demonstrating that the sufficient NADPH supply and well redox balance status are critical for the acetol production. Similar results were also observed in the *nadK*-overexpressing *E. coli* [29] and *C. glutamicum* [31], and *pntAB*-overexpressing *B. subtilis* [36]. The manipulations improved NADPH supply as well as the production of NADPH-dependent thymidine, l-isoleucine, and isobutanol, respectively. The NADH and NAD^+^ pools and the NADH/NAD^+^ ratio were gradually decreased from HJ06 to HJ06PN. Acetate formation was linked to the NADH/NAD^+^ ratio [41], which can explain the constantly reduction in this by-product formation from HJ06 to HJ06PN.

### Central carbon metabolism

Integration of gene expression profiles, metabolomics and fluxomics enabled a detailed picture of central carbon metabolism in acetol-producing strains. In the glycerol utilization pathway, concentrations of intracellular metabolites, and flux distribution were not differed in four strains, which are responsible for the similar glycerol consumption rate. This revealed the robustness of the metabolic network against genetic perturbations [42].

It is further seen that the flux values through the upper glycolytic pathway, the NADPH-generating PP pathway and the TCA cycle are nearly the same for HJ06 and HJ06N, and slightly changed in HJ06P and HJ06PN. This rigidity is not surprising, since the strains primarily use the carbon source for biosynthetic needs [43]. Sauer’s group [44] demonstrated that the relative flux into the PP pathway remained stable in 91 transcriptional regulator *E. coli* mutants on glucose and galactose. In addition, metabolites changes support the proposed flux scheme (Figs. 2 and 4). Furthermore, the rise in expression of genes *zwf* and *gnd* during the HJ06N/HJ06 comparison indicated that NADPH was limited under this condition (Fig. 5). Previous study found that *S. cerevisiae* responded to increasing NADPH requirement by the upregulation of genes in the oxidative PP pathway [45, 46]. The downregulation of these two genes during the HJ06P/HJ06 and HJ06PN/HJ06 comparisons was in agreement with the flux change (Figs. 2 and 5). This might be an indicative for enough NADPH supply, since transhydrogenase reaction was more activated to serve this role in *pntAB* overexpression strains. AcCoA diverges to the TCA cycle and the formation of acetate. Although the flux of carbon fueled to AcCoA from glycolysis differed among four strains, the end result was that optimized strains reduced acetate formation to keep the TCA cycle fluxes similar to HJ06. The flux pattern was consistent with transcriptional analysis of *gltA* (Figs. 2 and 5). The cells used the carbon for growth and energy, not for by-product formation, indicating the global flux flexibility towards growth and optimal flux distributions by resource allocation [47].

^13^C-MFA revealed the flexibility of the central metabolism at the DHAP node to accommodate acetol overproduction (Fig. 2). The carbon partitioning at this node directed gradually more carbon from the lower glycolytic pathway through the acetol biosynthetic pathway from HJ06 to HJ06PN. Since optimized strains can maintain similar fluxes through the upper glycolytic pathway as HJ06, the minority of the flux was diverted to the lower glycolytic pathway with less production of NADH (Fig. 4). The repression of *gapA* and *aceE* during the HJ06N/HJ06, HJ06P/HJ06 and HJ06PN/HJ06 comparisons (Fig. 5) correlated well with the decreased flux through these NADH-generating reactions (Fig. 2). The patterns of calculated flux through lower glycolysis were supported by measured changes in levels of GAP, 3PG, PEP, PYR and AcCoA (Fig. 4).

## Conclusions

^13^C-MFA-guided cofactor engineering combined with experimental validations provided useful information about the relevant targets for improving acetol production by engineered acetol-producing *E. coli* strains. Overexpression of *nadK* and *pntAB* either individually or in combination led to improvements in the acetol production. ^13^C-MFA illustrated that metabolism was rewired to reroute flux towards acetol production in optimized strains, which was supported by the most metabolomics data and gene expression profiles. This research demonstrated how ^13^C-MFA can be applied to drive the cycles of design, build, test and learn implementation for strain development.

## Methods

### Strains and culture conditions

*Escherichia coli* strains and plasmids are listed in Table 2. PCR was performed using the PrimeSTAR^®^ GXL DNA Polymerase (TaKaRa, Japan) and the appropriate primer pairs (Additional file 6). Each gene was assembled with the respective plasmid using In-Fusion Kit (TaKaRa, Japan). Plasmids were transformed into bacterial strains using electrotransformation. Where applicable, 50 µg/mL kanamycin was added to the media for selection.

**Table 2:** Strains and plasmids used in this study

Strains and plasmids	Relevant genotype or description	Source or reference
Strains		
*E. coli* BW25113	*F*^−^ *λ*^−^ *rph*^−*1*^ Δ*araBAD*_*AH33*_ *lacI*^*q*^ Δ*lacZ*_*WJ16*_ *rrnB*_*T14*_ Δ*rhaBAD*_*LD78*_ *hsdR514*	*E. coli* Genetic Stock Center from Yale University
*E. coli* Lin 43	Hfr(PO2A) *fhuA22*, *ΔphoA8*, *fadL701*(T2R), *relA1*, *glpR2*(*glp*^*c*^), *pitA10*, *spoT1*, *glpK22*(fbR), *rrnB*-*2*, *mcrB1*, *creC510*	*E. coli* Genetic Stock Center from Yale University
HJ02	BW25113 harboring pCA24 N-*yqhD*, *glpK* gene replaced by *glpK22* from strain Lin43	[22]
HJ06	HJ02 harboring pHN1009-*gapA*	This study
HJ06C	BW25113 harboring pHN1009-*gapA*, *glpK* gene replaced by *glpK22* from strain Lin43, *mgsA*^−^	This study
HJ06N	HJ06 harboring pBbB5K-*nadK*	This study
HJ06P	HJ06 harboring pBbB5K-*pntAB*	This study
HJ06PN	HJ06 harboring pBbB5K-*pntAB*-*nadK*	This study
Plasmids		
pCA24N	Cm; *lacI*^*q*^, pCA24N	[57]
pCA24N-*yqhD*	Cm; *lacI*^*q*^, pCA24N::*yqhD*^+^	[57]
pHN1009	pBR322 *ori*, *Amp*^*r*^, *lacI*^*q*^, P_trc_, *lac*^*o*^-PT-MCS	[58]
pHN1009-*gapA*	pHN1009 harboring *gapA* antisense sequence	[22]
pBbB5K-GFP	pBBR1;Kn^r^ *lacI* P_lac-UV5_	[59]
pBbB5K-*nadK*	Plasmid for *nadK* overexpression	This study
pBbB5K-*pntAB*	Plasmid for *pntAB* overexpression	This study
pBbB5K-*pntAB*-*nadK*	Plasmid for *pntAB* and *nadK* co-overexpression	This study

The pBbB5K-*nadK* and pBbB5K-*pntAB* were constructed as follows. The *nadK* and *pntAB* genes were amplified by PCR using the genome of *E*. *coli* BL21 as the template with the primer pairs nadk-F/nadk-R and pntAB-F/pntAB-R, respectively. The amplified fragments were cloned into the *Eco*RI/*Bam*HI site of pBbB5K, and the resulting plasmids were designated pBbB5K-*nadK* and pBbB5K-*pntAB*, respectively.

The cassette containing *pntAB* and *nadK* under control of the P_lacUV5_ promoter is constructed for further improving the production of acetol. Each gene was amplified using genome of *E*. *coli* BL21 as the templates and inserted to pBbB5K using In-Fusion kit. And the resulting plasmids were designated pBbB5K-*pntAB*-*nadK*. The plasmid was introduced into the strain HJ06, generating corresponding transformants. The *mgsA* gene was disrupted by P1 phage transduction [48].

The acetol-producing and non-producing *E. coli* strains were first precultured in LB medium for 8 h and then inoculated to (1% v/v) M9 minimal medium with 10 g/L glycerol. The cells were harvested at 12,000 rpm for 5 min, washed and inoculated into the main culture. The main culture containing 100 mL M9 with 10 g/L glycerol was incubated at 37 °C and shaken at 220 rpm in a 500 mL shake flask. For each strain, 11 flasks were used: three flasks were used for measurements of cell growth and extracellular metabolites, five flasks were used for intracellular metabolome analysis and qRT-PCR analysis at the mid-exponential growth phase, and three flasks were used for ^13^C-MFA at the mid-exponential growth phase. For ^13^C-labeling experiments, glycerol was added entirely as the [1,3-^13^C]-labeled isotope isomer (99%; Cambridge Isotope Laboratories, Andover, MA). If necessary, 50 µg/mL kanamycin was added to the media.

### Analytical methods

Cell density was monitored by measuring the optical density at 600 nm (OD_600_) of *E. coli* cultures. Glycerol, acetate, and acetol concentrations were determined by high-performance liquid chromatography (HPLC) (model 1260, Agilent, Santa Clara, USA) equipped with a refractive index detector as described previously [22].

### Sampling procedures for intracellular metabolome analysis and ^13^C-MFA

The quenching and extraction procedures for metabolome analysis and ^13^C-MFA were similar to the method described previously [49–51]. 10 mL culture was quenched in 5 mL 0.9% NaCl solution held at 0 °C in a liquid nitrogen bath. The mixtures were manually agitated to freeze the cells near 0 °C and centrifuged to remove the culture medium. To improve the precision of intracellular metabolite concentration measurements, we prepared uniformly ^13^C-labeled cell extract, which was used as internal standards (IS). The method was adapted from the studies of Wu et al. [52] and Weiner et al. [53] with slight modifications. For metabolite extraction, the cell pellets were suspended in 5 mL cold methanol containing IS. For ^13^C-MFA, IS was not added to the methanol. After sonication and vortexing with chloroform and Milli-Q water (72:28), the mixture was centrifuged for 5 min at 4600×*g* and 4 °C. The top layer was transferred into 5-kDa cutoff filters (Merck Millipore Ltd., Darmstadt, Germany) and then centrifuged to improve the extraction efficiency. The samples were completely lyophilized (FreeZone 6 Liter, Labconco, USA) and stored at − 80 °C.

### LC–MS analysis

Dried metabolite extracts were resuspended in 50 μL of methanol–water (1:1, v/v) and analyzed using a method as described previously [51]. Metabolite separation was achieved by a SeQuant ZIC-HILIC column (100 mm × 2.1 mm i.d., 3.5 µm) (Merck, Germany) using a Waters I-Class Acquity UPLC system (Waters, UK). The UPLC system was coupled to a Vion IMS QToF system (Waters, UK) [51].

Significant differences were analyzed by a two-tailed Student’s *t* test with Microsoft Excel 2016. The principal component analysis plots were generated using SIMCA 14.1 (Umetrics, Umea, Sweden). PCA was applied to the data after mean centering and UV scaling.

## ^13^C-MFA

The network model used for flux calculation was modified from our previous research [10] by excluding glucose metabolism and including the acetol biosynthetic pathway (Additional file 7). ^13^C-MFA was performed using the elementary metabolite unit (EMU)-based software, INCA [54, 55]. Fluxes were estimated by minimizing the lack-of-fit between experimentally measured and simulated mass isotopomer distributions of intracellular metabolites and the measured external rates, using least-squares regression. Each flux estimation was repeated a minimum of 100 times from random initial values to find a global solution. A *χ*^2^-statistical test for goodness-of-fit was performed. The nonlinear 95% confidence intervals were computed for all estimated fluxes by computing the sensitivity of the minimized variance-weighted sum of squared residuals to flux variations [56].

### qRT-PCR

RNA was extracted from mid-exponential growing cells to perform qRT-PCR as previously described [10]. The sequences of primer pairs are listed in Additional file 6. Significant differences were analyzed by one-way ANOVA using Microsoft Excel 2016.

## Additional files

**Additional file 1.** Metabolic pathways for acetol biosynthesis in *E. coli.*

**Additional file 2.** Measured and simulated mass isotopomer distributions of intracellular metabolites.

**Additional file 3.** Best fit-fluxes and flux confidence intervals.

**Additional file 4.** Goodness-of-fit analysis for ^13^C-MFA.

**Additional file 5.** Metabolomic data corresponding to the heat map in Fig. 4.

**Additional file 6.** Primers used in this study.

**Additional file 7.** Metabolic network model used for ^13^C-MFA.

## Abbreviations

AcCoA: acetyl-CoA
AKG: α-ketoglutarate
CIT: citrate
DHAP: dihydroxyacetone phosphate
E4P: erythrose 4-phosphate
F6P: fructose 6-phosphate
FBP: fructose 1,6-bisphosphate
FUM: fumarate
G6P: glucose-6-phosphate
GAP: glyceraldehyde 3-phosphate
GLX: glyoxylate
Gly3P: glycerol-3-phosphate
ICIT: isocitrate
MAL: malate
OAA: oxaloacetate
3PG: 3-phosphoglyceric acid
6PG: 6-phosphogluconate
PEP: phosphoenolpyruvate
PYR: pyruvate
R5P: ribose 5-phosphate
RU5P: ribulose 5-phosphate
S7P: sedoheptulose 7-phosphate
SUCCoA: succinyl-CoA
SUC: succinate
X5P: xylulose 5-phosphate
