# Supplementary figures and images for: 13C metabolic flux analysis-guided metabolic engineering of Escherichia coli for improved acetol production from glycerol
Source: Biotechnol Biofuels. 2019 Feb 13;12:29. doi: 10.1186/s13068-019-1372-4 (PMC6373095; doi:10.1186/s13068-019-1372-4)

**Additional file 1** Metabolic pathways for acetol biosynthesis in *E. coli*.

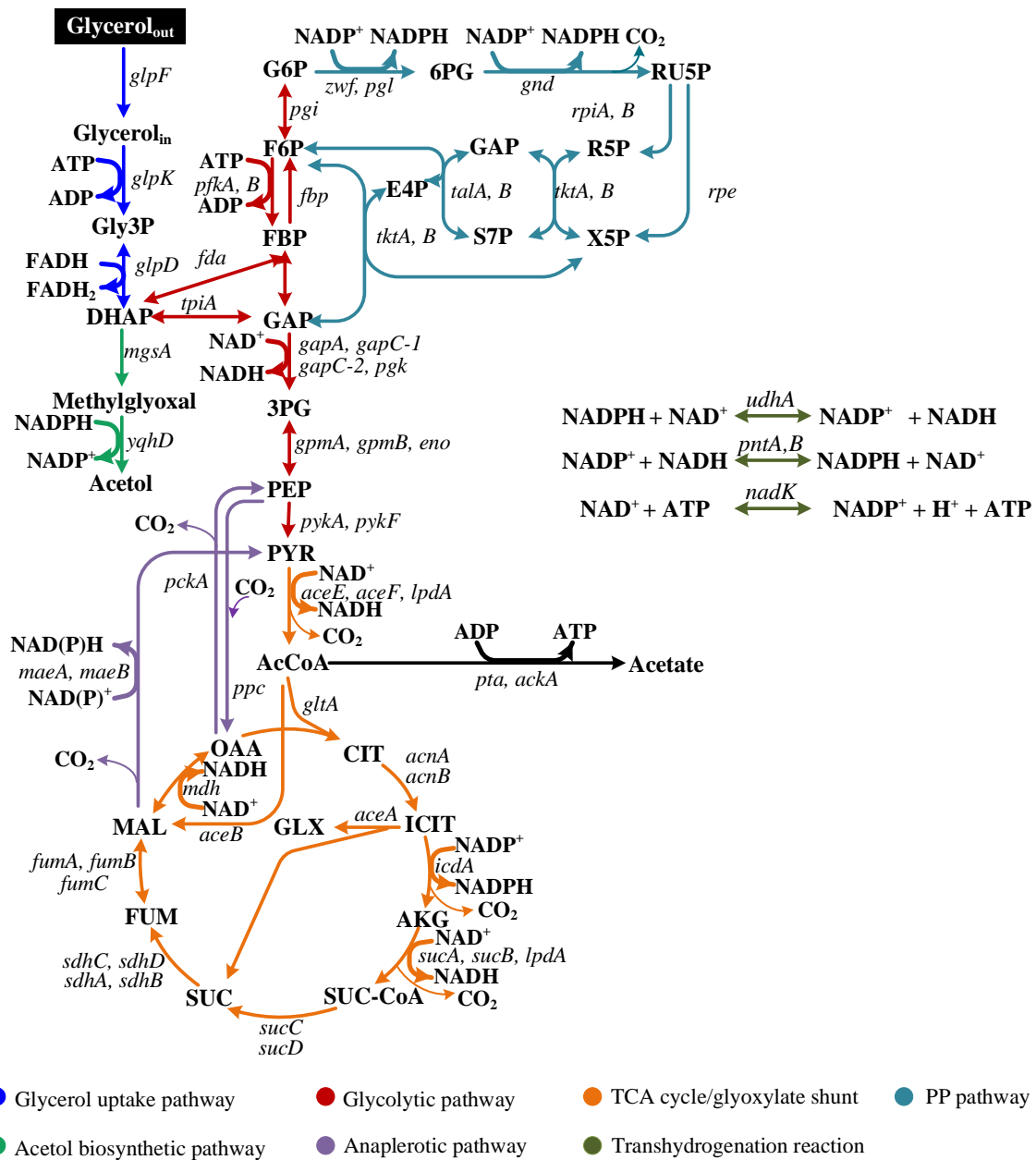

Supplement: Supplementary file 1 — Additional file 1. Metabolic pathways for acetol biosynthesis in E. coli. [file 13068_2019_1372_MOESM1_ESM.pdf]
