# Supplementary material for: 13C metabolic flux analysis-guided metabolic engineering of Escherichia coli for improved acetol production from glycerol
Source: Biotechnol Biofuels. 2019 Feb 13;12:29. doi: 10.1186/s13068-019-1372-4 (PMC6373095; doi:10.1186/s13068-019-1372-4)
Supplement: Supplementary file 2 — Additional file 2. Measured and simulated mass isotopomer distributions of intracellular metabolites. [file 13068_2019_1372_MOESM2_ESM.pdf]

Additional file 2. Measured and simulated mass isotopomer distributions of intracellular metabolites.

		HJ06		HJ06C		HJ06N		HJ06P		HJ06PN	
Substance	Mass isotopomer	Abundance (normalized to 100)									
		Measured	Simulated	Measured	Simulated	Measured	Simulated	Measured	Simulated	Measured	Simulated
Gly3P	M	0.0±0.1	0.0	0.0±0.1	0.0	0.0±0.1	0.0	0.0±0.1	0.0	0.0±0.1	0.0
	M+1	2.2±0.1	2.1	2.2±0.1	2.1	2.2±0.1	2.1	2.4±0.1	2.1	2.4±0.1	2.3
	M+2	97.8±0.3	97.8	97.8±0.3	97.8	97.8±0.3	97.8	97.6±0.3	97.8	97.5±0.3	97.6
	M+3	0.0±0.1	0.1	0.0±0.1	0.1	0.0±0.1	0.1	0.0±0.1	0.1	0.0±0.1	0.2
DHAP	M	0.1±0.1	0.1	0.1±0.1	0.1	0.1±0.1	0.0	0.0±0.1	0.0	0.0±0.1	0.0
	M+1	2.9±0.1	3.3	3.1±0.1	2.9	2.2±0.1	2.3	2.3±0.1	2.1	2.3±0.1	2.4
	M+2	96.2±0.3	95.6	96.6±0.3	96.5	97.7±0.3	97.4	97.7±0.3	97.8	97.6±0.3	97.4
	M+3	0.9±0.1	1.0	0.2±0.1	0.6	0.0±0.1	0.2	0.0±0.1	0.1	0.0±0.1	0.2
GAP	M	0.1±0.1	0.1	0.1±0.1	0.1	0.0±0.1	0.0	0.0±0.1	0.0	0.0±0.1	0.0
	M+1	2.9±0.1	3.3	3.0±0.1	2.9	2.3±0.1	2.3	2.4±0.1	2.1	2.5±0.1	2.4
	M+2	96.1±0.3	95.6	95.7±0.3	96.5	97.7±0.3	97.4	97.6±0.3	97.8	97.5±0.3	97.4
	M+3	0.8±0.1	1.0	1.1±0.1	0.6	0.0±0.1	0.2	0.0±0.1	0.1	0.0±0.1	0.2
FBP	M	0.0±0.1	0.0	0.0±0.1	0.0	0.0±0.1	0.0	0.0±0.1	0.0	0.0±0.1	0.0
	M+1	0.0±0.1	0.0	0.0±0.1	0.0	0.0±0.1	0.0	0.0±0.1	0.0	0.0±0.1	0.0
	M+2	0.2±0.1	0.3	0.3±0.1	0.4	0.1±0.1	0.1	0.1±0.1	0.1	0.1±0.1	0.1
	M+3	5.9±0.1	6.4	7.2±0.1	6.6	4.8±0.1	4.6	4.9±0.1	4.6	5.1±0.1	4.6
	M+4	92.3±0.3	91.4	91.3±0.3	91.4	94.8±0.3	94.9	94.9±0.3	94.9	94.7±0.3	94.9
	M+5	1.6±0.1	1.9	1.2±0.1	1.7	0.3±0.1	0.4	0.0±0.1	0.4	0.0±0.1	0.4
	M+6	0.0±0.1	0.0	0.0±0.1	0.0	0.0±0.1	0.0	0.0±0.1	0.0	0.0±0.1	0.0
G6P	M	0.1±0.1	0.0	0.0±0.1	0.0	0.0±0.1	0.0	0.0±0.1	0.0	0.0±0.1	0.0
	M+1	0.2±0.1	0.1	0.1±0.1	0.1	0.1±0.1	0.1	0.1±0.1	0.1	0.1±0.1	0.1
	M+2	2.6±0.1	1.8	2.1±0.1	2.0	2.4±0.1	2.4	2.6±0.1	2.4	2.7±0.1	2.3
	M+3	20.3±0.2	20.5	21.8±0.2	21.3	23.7±0.2	24.4	24.2±0.2	24.4	24.4±0.2	24.9
	M+4	63.8±0.3	64.4	64.5±0.3	65.3	62.8±0.3	61.7	61.9±0.3	61.8	61.4±0.3	61.6
	M+5	12.8±0.1	13.0	11.4±0.1	11.3	10.9±0.1	11.3	11.2±0.1	11.3	11.5±0.1	11.1

	M+6	0.2±0.1	0.1	0.0±0.1	0.1	0.0±0.1	0.0	0.0±0.1	0.0	0.0±0.1	0.0
F6P	M	0.1±0.1	0.0	0.1±0.1	0.0	0.0±0.1	0.0	0.0±0.1	0.0	0.0±0.1	0.0
	M+1	0.4±0.1	0.1	0.2±0.1	0.1	0.1±0.1	0.1	0.1±0.1	0.1	0.1±0.1	0.1
	M+2	2.3±0.1	1.8	2.0±0.1	2.0	2.3±0.1	2.4	2.4±0.1	2.4	2.5±0.1	2.3
	M+3	19.9±0.1	20.5	21.8±0.1	21.3	23.7±0.2	24.4	24.4±0.2	24.4	24.7±0.2	24.9
	M+4	64±0.3	64.4	64.6±0.3	65.3	62.9±0.3	61.7	61.6±0.3	61.8	61±0.3	61.6
	M+5	13.2±0.1	13.0	11.3±0.1	11.3	11.0±0.1	11.3	11.5±0.1	11.3	11.8±0.1	11.1
	M+6	0.2±0.1	0.1	0.0±0.1	0.1	0.0±0.1	0.0	0.0±0.1	0.0	0.0±0.1	0.0
R5P	M	0.0±0.1	0.0	0.0±0.1	0.0	0.0±0.1	0.0	0.0±0.1	0.0	0.0±0.1	0.0
	M+1	0.5±0.1	0.3	0.3±0.1	0.3	0.3±0.1	0.3	0.4±0.1	0.3	0.4±0.1	0.3
	M+2	9.1±0.1	9.2	10.3±0.1	10.8	13.8±0.1	13.5	13.6±0.1	13.6	14.0±0.1	14.0
	M+3	69.4±0.3	69.2	64.2±0.3	63.3	57±0.3	56.9	56.6±0.3	56.6	56.2±0.3	56.4
	M+4	20.8±0.2	21.1	25.1±0.2	25.5	28.8±0.2	29.3	29.5±0.2	29.5	29.5±0.2	29.2
	M+5	0.2±0.1	0.2	0.1±0.1	0.1	0.1±0.1	0.1	0.0±0.1	0.0	0.0±0.1	0.1
3PG	M	0.1±0.1	0.1	0.1±0.1	0.1	0.0±0.1	0.0	0.0±0.1	0.0	0.0±0.1	0.0
	M+1	4.4±0.1	3.6	2.7±0.1	2.9	3.0±0.1	2.4	3.3±0.1	3.0	3.5±0.1	2.5
	M+2	94.4±0.3	95.2	96.9±0.3	96.5	96.9±0.3	97.3	96.7±0.3	96.6	96.5±0.3	97.1
	M+3	1.1±0.1	1.1	0.2±0.1	0.6	0.0±0.1	0.2	0.0±0.1	0.4	0.0±0.1	0.3
PEP	M	0.1±0.1	0.1	0.1±0.1	0.1	0.1±0.1	0.0	0.0±0.1	0.0	0.0±0.1	0.0
	M+1	4.1±0.1	3.6	2.2±0.1	2.9	2.8±0.1	2.4	2.9±0.1	3.1	3.1±0.1	2.5
	M+2	94.5±0.3	95.2	97.4±0.3	96.5	97.1±0.3	97.3	97.1±0.3	96.4	96.9±0.3	97.1
	M+3	1.3±0.1	1.1	0.2±0.1	0.6	0.0±0.1	0.2	0.0±0.1	0.4	0.0±0.1	0.3
PYR	M	0.2±0.1	0.1	0.1±0.1	0.1	0.0±0.1	0.0	0.0±0.1	0.0	0.0±0.1	0.0
	M+1	3.7±0.1	3.6	3.6±0.1	2.9	3.7±0.1	2.4	4.0±0.1	3.1	3.1±0.1	2.5
	M+2	95.0±0.3	95.2	96.0±0.3	96.5	96.3±0.3	97.3	95.5±0.3	96.4	96.6±0.3	97.1
	M+3	1.2±0.1	1.1	0.3±0.1	0.6	0.1±0.1	0.2	0.5±0.1	0.4	0.2±0.1	0.3
Cit	M	0.0±0.1	0.0	0.0±0.1	0.0	0.0±0.1	0.0	0.0±0.1	0.0	0.0±0.1	0.0
	M+1	0.1±0.1	0.0	0.1±0.1	0.0	0.0±0.1	0.0	0.1±0.1	0.0	0.1±0.1	0.0
	M+2	2.5±0.1	2.4	2.3±0.1	2.1	1.8±0.1	2.0	2.4±0.1	2.1	1.9±0.1	1.8

	M+3	47.3±0.2	47.6	49.8±0.2	48.4	47.7±0.2	47.4	47.7±0.2	48.9	48.3±0.2	49.2
	M+4	48.8±0.2	48.5	47.6±0.2	48.6	49.8±0.3	48.8	48.7±0.2	47.4	49.2±0.2	48.4
	M+5	1.3±0.1	1.5	0.2±0.1	0.8	0.6±0.1	1.7	1.1±0.1	1.6	0.5±0.1	0.6
	M+6	0.0±0.1	0.0	0.0±0.1	0.0	0.0±0.1	0.0	0.0±0.1	0.0	0.0±0.1	0.0
AKG	M	0.0±0.1	0.0	0.0±0.1	0.0	0.0±0.1	0.0	0.0±0.1	0.0	0.0±0.1	0.0
	M+1	0.9±0.1	0.7	0.7±0.1	0.6	0.6±0.1	0.5	0.6±0.1	0.6	0.6±0.1	0.5
	M+2	25.0±0.2	24.2	25.2±0.2	23.9	26.0±0.2	24.6	26.0±0.2	25.7	25.9±0.2	22.6
	M+3	66.1±0.3	67.1	67.5±0.3	68	66.6±0.3	67.4	66.1±0.3	65.9	64.7±0.3	65.0
	M+4	7.9±0.1	8.0	6.7±0.1	7.4	6.8±0.1	7.3	7.2±0.1	7.7	8.8±0.1	11.8
	M+5	0.1±0.1	0.1	0.0±0.1	0.1	0.1±0.1	0.2	0.1±0.1	0.2	0.1±0.1	0.1
SUC	M	0.0±0.1	0.0	0.0±0.1	0.0	0.0±0.1	0.0	0.0±0.1	0.0	0.0±0.1	0.0
	M+1	3.0±0.1	2.7	2.4±0.1	2.3	2.1±0.1	2.5	2.3±0.1	2.4	1.9±0.1	1.6
	M+2	72.6±0.3	73.4	71.8±0.3	73.9	74.9±0.3	75.5	73.4±0.3	74.8	68.6±0.3	64.1
	M+3	24.1±0.2	23.5	25.5±0.2	23.5	22.7±0.2	21.4	24.0±0.2	22.3	29.3±0.2	34.1
	M+4	0.3±0.1	0.4	0.3±0.1	0.2	0.3±0.1	0.5	0.4±0.1	0.5	0.2±0.1	0.2
Fum	M	0.1±0.1	0.0	0.1±0.1	0.0	0.0±0.1	0.0	0.0±0.1	0.0	0.0±0.1	0.0
	M+1	2.7±0.1	2.6	2.4±0.1	2.3	2.0±0.1	2.4	2.2±0.1	2.3	1.8±0.1	1.6
	M+2	69.8±0.3	70.3	71.3±0.3	71.8	73.3±0.3	73.4	72.1±0.3	72.2	67.6±0.3	62.6
	M+3	27.0±0.2	26.7	26.0±0.2	25.7	24.4±0.2	23.6	25.3±0.2	25.0	30.4±0.2	35.6
	M+4	0.4±0.1	0.4	0.3±0.1	0.3	0.2±0.1	0.5	0.4±0.1	0.5	0.2±0.1	0.2
Mal	M	0.0±0.1	0.0	0.0±0.1	0.0	0.0±0.1	0.0	0.0±0.1	0.0	0.0±0.1	0.0
	M+1	2.8±0.1	2.6	2.3±0.1	2.3	2.1±0.1	2.4	2.1±0.1	2.3	1.7±0.1	1.6
	M+2	70.4±0.3	70.3	71.8±0.3	71.7	73.9±0.3	73.2	72.6±0.3	72.2	68.2±0.3	62.6
	M+3	26.4±0.2	26.7	25.8±0.2	25.8	23.7±0.2	23.8	24.9±0.2	25.0	29.9±0.2	35.6
	M+4	0.4±0.1	0.4	0.1±0.1	0.3	0.2±0.1	0.5	0.4±0.1	0.5	0.2±0.1	0.2
Glu	M	0.0±0.1	0.0	0.0±0.1	0.0	0.0±0.1	0.0	0.0±0.1	0.0	0.0±0.1	0.0
	M+1	0.8±0.1	0.7	0.7±0.1	0.6	0.7±0.1	0.5	0.6±0.1	0.6	0.6±0.1	0.5
	M+2	25.1±0.2	24.2	25.4±0.2	23.9	25.7±0.2	24.6	27.1±0.2	25.7	26.3±0.2	22.6
	M+3	66.3±0.3	67.1	67.5±0.3	68	66.8±0.3	67.4	64.7±0.3	65.9	64.0±0.3	65.0

	M+4	7.8±0.1	8.0	6.3±0.1	7.4	6.7±0.1	7.3	7.5±0.1	7.7	9.1±0.1	11.8
	M+5	0.1±0.1	0.1	0.0±0.1	0.1	0.1±0.1	0.2	0.1±0.1	0.2	0.1±0.1	0.1
Asp	M	0.0±0.1	0.0	0.0±0.1	0.0	0.0±0.1	0.0	0.0±0.1	0.0	0.0±0.1	0.0
	M+1	1.9±0.1	1.8	1.7±0.1	1.5	1.4±0.1	1.5	1.5±0.1	1.6	1.4±0.1	1.2
	M+2	47.7±0.2	48.4	49.2±0.2	48.9	48.8±0.2	48.7	49.8±0.2	50.1	49.6±0.2	49.6
	M+3	49.7±0.2	49.2	49.1±0.2	49.2	49.6±0.3	49.4	48.4±0.2	47.9	48.8±0.2	49.0
	M+4	0.6±0.1	0.7	0.0±0.1	0.3	0.2±0.1	0.4	0.3±0.1	0.4	0.1±0.1	0.2
Ala	M	0.1±0.1	0.1	0.2±0.1	0.1	0.0±0.1	0.0	0.0±0.1	0.0	0.0±0.1	0.0
	M+1	4.0±0.1	3.6	3.7±0.1	2.9	3±0.1	2.4	4.2±0.1	3.1	3.4±0.1	2.5
	M+2	94.6±0.3	95.2	95.9±0.3	96.5	96.9±0.3	97.3	95.3±0.3	96.4	96.4±0.3	97.1
	M+3	1.2±0.1	1.1	0.3±0.1	0.6	0.1±0.1	0.2	0.5±0.1	0.4	0.2±0.1	0.3
Phe	M	0.0±0.1	0.0	0.0±0.1	0.0	0.0±0.1	0.0	0.0±0.1	0.0	0.0±0.1	0.0
	M+1	0.0±0.1	0.0	0.0±0.1	0.0	0.0±0.1	0.0	0.0±0.1	0.0	0.0±0.1	0.0
	M+2	0.0±0.1	0.0	0.0±0.1	0.0	0.0±0.1	0.0	0.0±0.1	0.0	0.0±0.1	0.0
	M+3	0.1±0.1	0.1	0.1±0.1	0.1	0.0±0.1	0.1	0.0±0.1	0.1	0.0±0.1	0.1
	M+4	2.2±0.1	1.9	1.8±0.1	1.7	1.5±0.1	1.5	1.5±0.1	1.6	1.6±0.1	1.5
	M+5	23.6±0.2	24.1	24.9±0.2	25.6	26.5±0.2	27.0	27.2±0.2	27.0	27.6±0.2	26.5
	M+6	71.1±0.3	71.1	72.0±0.3	71.3	71.9±0.3	70.9	71.2±0.3	70.0	70.8±0.3	71.1
	M+7	2.9±0.1	2.8	1.1±0.1	1.4	0.1±0.1	0.6	0.0±0.1	1.3	0.0±0.1	0.8
	M+8	0.0±0.1	0.0	0.0±0.1	0.0	0.0±0.1	0.0	0.0±0.1	0.0	0.0±0.1	0.0
	M+9	0.0±0.1	0.0	0.0±0.1	0.0	0.0±0.1	0.0	0.0±0.1	0.0	0.0±0.1	0.0
Leu	M	0.0±0.1	0.0	0.0±0.1	0.0	0.0±0.1	0.0	0.0±0.1	0.0	0.0±0.1	0.0
	M+1	0.1±0.1	0.1	0.1±0.1	0.0	0.1±0.1	0.0	0.1±0.1	0.0	0.1±0.1	0.0
	M+2	4.1±0.1	3.9	3.4±0.1	3.6	3.5±0.1	3.2	3.6±0.1	3.1	3.8±0.1	3.2
	M+3	91.7±0.3	91.2	93.5±0.3	94.0	94.5±0.3	93.5	91.1±0.3	92.0	94.1±0.3	94.9
	M+4	4.0±0.1	4.8	3.0±0.1	2.4	1.9±0.1	3.3	5.1±0.1	4.9	2.0±0.1	1.9
	M+5	0.0±0.1	0.1	0.0±0.1	0.0	0.0±0.1	0.0	0.1±0.1	0.1	0.0±0.1	0.0
	M+6	0.0±0.1	0.0	0.0±0.1	0.0	0.0±0.1	0.0	0.0±0.1	0.0	0.0±0.1	0.0
Ile	M	0.0±0.1	0.0	0.0±0.1	0.0	0.0±0.1	0.0	0.0±0.1	0.0	0.0±0.1	0.0

	M+1	0.1±0.1	0.0	0.1±0.1	0.0	0.1±0.1	0.0	0.0±0.1	0.0	0.0±0.1	0.0
	M+2	3.0±0.1	2.4	2.4±0.1	2.1	2.0±0.1	2.1	2.1±0.1	2.1	1.8±0.1	1.8
	M+3	45.7±0.2	47.6	49.4±0.2	48.6	48.8±0.2	48.5	49.1±0.2	49.5	49.8±0.2	49.2
	M+4	49.6±0.2	48.5	48.1±0.2	48.6	48.8±0.2	48.8	47.6±0.2	47.4	47.9±0.2	48.4
	M+5	1.6±0.1	1.5	0.1±0.1	0.7	0.4±0.1	0.6	1.1±0.1	1.1	0.5±0.1	0.6
	M+6	0.0±0.1	0.0	0.0±0.1	0.0	0.0±0.1	0.0	0.0±0.1	0.0	0.0±0.1	0.0
Met	M	0.0±0.1	0.0	0.0±0.1	0.0	0.0±0.1	0.0	0.0±0.1	0.0	0.0±0.1	0.0
	M+1	1.0±0.1	0.9	1.0±0.1	0.8	0.8±0.1	0.9	0.8±0.1	0.8	0.7±0.1	0.6
	M+2	26.6±0.2	25.1	27.3±0.2	25.9	28.5±0.2	30.1	26.4±0.2	25.5	26.6±0.2	24.5
	M+3	47.1±0.2	48.8	48.3±0.2	49.1	47.3±0.2	49.1	47.2±0.2	49.0	46.9±0.2	49.3
	M+4	25.0±0.2	24.9	23.3±0.2	24.1	23.4±0.2	19.7	25.5±0.2	24.5	25.7±0.2	25.5
	M+5	0.3±0.1	0.3	0.1±0.1	0.2	0.1±0.1	0.2	0.1±0.1	0.2	0.1±0.1	0.1
Ser	M	0.1±0.1	0.1	0.1±0.1	0.1	0.0±0.1	0.1	0.0±0.1	0.0	0.0±0.1	0.1
	M+1	3.7±0.1	3.7	3.8±0.1	3.3	2.2±0.1	2.8	3.0±0.1	3.0	3.3±0.1	2.7
	M+2	94.9±0.3	94.9	95.7±0.3	95.7	97.7±0.3	96.8	96.9±0.3	96.6	96.7±0.3	96.8
	M+3	1.2±0.1	1.2	0.4±0.1	0.9	0.1±0.1	0.3	0.0±0.1	0.4	0.0±0.1	0.4
Gln	M	0.0±0.1	0.0	0.0±0.1	0.0	0.0±0.1	0.0	0.0±0.1	0.0	0.0±0.1	0.0
	M+1	0.9±0.1	0.7	0.8±0.1	0.6	0.6±0.1	0.5	0.7±0.1	0.6	0.7±0.1	0.5
	M+2	25.1±0.2	24.2	25.1±0.2	23.9	25.5±0.2	24.6	27±0.2	25.7	26.2±0.2	22.6
	M+3	66.1±0.3	67.1	67.7±0.3	68.0	66.9±0.3	67.4	64.7±0.3	65.9	64.0±0.3	65.0
	M+4	7.8±0.1	8.0	6.4±0.1	7.4	7.0±0.1	7.3	7.4±0.1	7.7	9.0±0.1	11.8
	M+5	0.1±0.1	0.1	0.0±0.1	0.1	0.1±0.1	0.2	0.1±0.1	0.2	0.1±0.1	0.1

Measured: values represent the mean ± SD of three independent samples, which are corrected mass isotopomer abundances.

Simulated: values simulated by the isotope model.
