# Supplementary material for: 13C metabolic flux analysis-guided metabolic engineering of Escherichia coli for improved acetol production from glycerol
Source: Biotechnol Biofuels. 2019 Feb 13;12:29. doi: 10.1186/s13068-019-1372-4 (PMC6373095; doi:10.1186/s13068-019-1372-4)
Supplement: Supplementary file 4 — Additional file 4. Goodness-of-fit analysis for 13C-MFA. [file 13068_2019_1372_MOESM4_ESM.pdf]

Additional file 4. Goodness-of-fit analysis for ^{13}C-MFA.

Strains	Degrees of freedom	95% Confidence Interval LB (SSR)	95% Confidence Interval UB (SSR)	Sum of Squared Residuals (SSR)	Acceptance
HJ06	61	41.3	84.5	52.4	Yes
HJ06C	63	43.0	86.8	78.8	Yes
HJ06N	61	41.3	84.5	43.8	Yes
HJ06P	61	41.3	84.5	73.8	Yes
HJ06PN	61	41.3	84.5	81.6	Yes

LB=lower bound, UB=upper bound.
