# Supplementary material for: 13C metabolic flux analysis-guided metabolic engineering of Escherichia coli for improved acetol production from glycerol
Source: Biotechnol Biofuels. 2019 Feb 13;12:29. doi: 10.1186/s13068-019-1372-4 (PMC6373095; doi:10.1186/s13068-019-1372-4)
Supplement: Supplementary file 6 — Additional file 6. Primers used in this study. [file 13068_2019_1372_MOESM6_ESM.pdf]

Additional file 6 Primers used in this study.

Primer	Sequence
Primers for gene overexpression	
nadK-F	ATAACAATTTCAGAATTCATGAATAATCATTTCAAGTGTATTG
nadK-R	CCTTACTCGAGTTTGGATCCTTAGAATAATTTTTTTGACCAGCCG
pntAB-F	ATAACAATTTCAGAATTC ATGCGAATTGGCATACCAAGAGAAC
pntAB-R	CCTTACTCGAGTTTGGATCC TTACAGAGCTTTCAGGATTGCATCC
Infusion	
pntAB-F	ATAACAATTTCAGAATTC ATGCGAATTGGCATACCAAGAGAAC
pntAB-R	TTACAGAGCTTTCAGGATTGCATCC
nadK-F	GGATGCAATCCTGAAAGCTCTGTAA AATTCGGCACGAGCTGCAACGAAAGCATGAATAATCATTTCAAGTGTATTG
nadK-R	CCTTACTCGAGTTTGGATCC TTAGAATAATTTTTTTGACCAGCCG
Primers for qRT-PCR	
glpK-F	TAACGACGCCTACGATTCCG
glpK-R	CCCACGAGTCAGACCGAAAA
glpD-F	CGACCTGAAACTGGACTCGC
glpD-R	ACGATGCGCTGGTCTTCATT
pntA-F	GCGGAAATGGAACTCTTTGC
pntB-R	TTCACGGGTAATTAGCTTCGG
zwf-F	AGTCATGGAGAAACCGCTGG
zwf-R	GGGAGTTAGCAAAACGCAGC
gnd-F	GAAAGATCAGCGTGTTGCCG
gnd-R	CGCCGTAGTTCAGATCCCAG
icdA-F	ACACTGGCAAAGAGATCGTC
icdA-R	CAGGGCGTCAGAAATGTAGTC
aceE-F	TGAAGACCGCGCTCATTACC
aceE-R	CGGAATGGTGTTGCGATAGG
gltA-F	ACTCTCGGTTCAAAAGGTGTG
gltA-R	GTAGTTAGAATCGGTCGCCAG
pta-F	ACAATGTTGATCCGGCGAAG
pta-R	CATATCGATCGCACGAGTCG
ackA-F	CTGGTTCTGAACTGCGGTAGTTC
ackA-R	GGCAGGTGGAAACATTCGG
maeB-F	AGTTCCAGGGAAAATCCAGG
maeB-R	CCTCGGGCGGTATATTTGTAG
nadK-F	ACGTTGTCAGCACGACCACTG
nadK-R	GGCTGTCGCAACTGATTTCCAG
gapA-F	TCCGACCCCGAACGTATCTGTA
gapA-R	CTTCAGCAGCAGCTTTAACGGC
sucA-F	AAGTGATTCCGTTTGGGGCTTC
sucA-R	TGCTGTTTCTGGTGAACGGACA
