# Supplementary material for: 13C metabolic flux analysis-guided metabolic engineering of Escherichia coli for improved acetol production from glycerol
Source: Biotechnol Biofuels. 2019 Feb 13;12:29. doi: 10.1186/s13068-019-1372-4 (PMC6373095; doi:10.1186/s13068-019-1372-4)
Supplement: Supplementary file 7 — Additional file 7. Metabolic network model used for 13C-MFA. [file 13068_2019_1372_MOESM7_ESM.pdf]

Additional file 7 Metabolic network model used for ^{13}C-MFA

**Glycerol Metabolism**	
v1	Glyc (abc) + ATP -> Glyc3P (abc)
v2	Glyc3P (abc) <-> DHAP (abc) + $FADH_2$
**Glycolysis**	
v3	F6P (abcdef) <-> G6P (abcdef)
v4	FBP (abcdef) <-> F6P (abcdef) + ATP
v5	FBP (abcdef) <-> DHAP (cba) + GAP (def)
v6	DHAP (abc) <-> GAP (abc)
v7	GAP (abc) -> 3PG (abc) + NADH + ATP
v8	3PG (abc) <-> PEP (abc)
v9	PEP (abc) -> Pyr (abc) + ATP
**Pentose Phosphate Pathway**	
v10	G6P (abcdef) -> 6PG (abcdef) + NADPH
v11	6PG (abcdef) -> Ru5P (bcdef) + CO_2 (a) + NADPH
v12	Ru5P (abcde) <-> X5P (abcde)
v13	Ru5P (abcde) <-> R5P (abcde)
v14	X5P (abcde) + R5P (fghij) <-> S7P (abfghij) + GAP (cde)
v15	X5P (abcde) + E4P (fghi) <-> F6P (abfghi) + GAP (cde)
v16	GAP (hij) + S7P (abcdefg) <-> F6P (abchij) + E4P (defg)
**TCA Cycle**	
v17	Pyr (abc) -> AcCoA (bc) + CO_2 (a) + NADH
v18	OAA (abcd) + AcCoA (ef) -> Cit (dcbfea)
v19	Cit (abcdef) -> ICit (abcdef)
v20	ICit (abcdef) -> AKG (abcde) + CO_2 (f) + NADPH
v21	AKG (abcde) -> SucCoA (bcde) + CO_2 (a) + NADH
v22	SucCoA (abcd) -> Suc (abcd) + ATP
v23	Suc (abcd) -> Fum (abcd) + $FADH_2$
v24	Fum (abcd) <-> Mal (abcd)
v25	Mal (abcd) <-> OAA (abcd) + NADH
**Glyoxylate Shunt**	
v26	ICit (abcdef) + AcCoA (gh) -> Mal (abhg) + Suc (fcde)
**Acetic Acid Formation**	
v27	AcCoA (ab) -> Acetate (ab) + ATP
**Acetol Production Pathway**	
v28	DHAP (abc) -> Methylglyoxal (abc)

v29 Methylglyoxal (abc) + NADPH -> Acetol (abc)

Amphibolic Reactions

v30 Mal (abcd) -> Pyr (abc) + CO_2 (d) + 0.5*NADPH+ 0.5*NADH

v31 PEP (abc) + CO_2 (d) -> OAA (abcd)

v32 OAA (abcd) + ATP -> PEP (abc) + CO2 (d)

Oxidative Phosphorylation

v33 NADH + 0.5*O_2 -> 3*ATP

v34 FADH2 + 0.5*O_2 -> 2*ATP

Transhydrogenation

v35 NADPH <-> NADH

ATP Hydrolysis

v36 ATP -> ATP.ext

Transport

v37 Glyc.ext (abc) -> Glyc (abc)

v38 CO_2 (a) -> CO_2.ext (a)

v39 Acetate (ab) -> Acetate.ext (ab)

v40 Acetol (abc) -> Acetol.ext (abc)

v41 O_2.ext -> O_2

Biomass Formation

v42 0.488*Ala + 0.281*Arg + 0.229*Asn + 0.229*Asp + 0.087*Cys + 0.25*Glu + 0.25*Gln + 0.582*Gly + 0.09*His + 0.276*Ile + 0.428*Leu + 0.326*Lys + 0.146*Met + 0.176*Phe + 0.21*Pro + 0.205*Ser + 0.241*Thr + 0.054*Trp + 0.131*Tyr + 0.402*Val + 0.205*G6P + 0.071*F6P + 0.754*R5P + 0.129*GAP + 0.619*3PG + 0.051*PEP + 0.083*Pyr + 2.51*AcCoA + 0.087*AKG + 0.34*OAA + 0.443*MEETHF + 33.25*ATP + 5.363*NADPH -> 39.68*Biomass + 1.455*NADH

Amino Acid Biosynthesis

v43 AKG (abcde) + NADPH -> Glu (abcde)

v44 Glu (abcde) + ATP -> Gln (abcde)

v45 Glu (abcde) + ATP + 2*NADPH -> Pro (abcde)

v46 Glu (abcde) + CO_2 (f) + Gln (ghijk) + Asp (lmno) + 5*ATP + NADPH -> Arg (abcdef) + AKG (ghijk) + Fum (lmno)

v47 OAA (abcd) + Glu (efghi) -> Asp (abcd) + AKG (efghi)

v48 Asp (abcd) + 2*ATP -> Asn (abcd)

v49 Pyr (abc) + Glu (defgh) -> Ala (abc) + AKG (defgh)

v50 3PG (abc) + Glu (defgh) -> Ser (abc) + AKG (defgh) + NADH

v51 Ser (abc) <-> Gly (ab) + MEETHF (c)

v52 Gly (ab) <-> CO_2 (a) + MEETHF (b) + NADH

v53 Thr (abcd) -> Gly (ab) + AcCoA (cd) + NADH

v54 Ser (abc) + 3*ATP + 4*NADPH -> Cys (abc)

v55 Asp (abcd) + Pyr (efg) + Glu (hijkl) + SucCoA (mnop) + ATP + 2*NADPH -> LL_DAP (abcdgfe) + AKG (hijkl) + Suc (mnop)

v56	LL_DAP (abcdefg) -> Lys (abcdef) + CO_2 (g)
v57	Asp (abcd) + 2*ATP + 2*NADPH -> Thr (abcd)
v58	Asp (abcd) + METHF (e) + Cys (fgh) + SucCoA (ijkl) + ATP + 2*NADPH -> Met (abcde) + Pyr (fgh) + Suc (ijkl)
v59	Pyr (abc) + Pyr (def) + Glu (ghijk) + NADPH -> Val (abcef) + CO_2 (d) + AKG (ghijk)
v60	AcCoA (ab) + Pyr (cde) + Pyr (fgh) + Glu (ijklm) + NADPH -> Leu (abdghe) + CO_2 (c) + CO_2 (f) + AKG (ijklm) + NADH
v61	Thr (abcd) + Pyr (efg) + Glu (hijkl) + NADPH -> Ile (abfcdg) + CO_2 (e) + AKG (hijkl)
v62	PEP (abc) + PEP (def) + E4P (ghij) + Glu (klmno) + ATP + NADPH -> Phe (abcefghij) + CO_2 (d) + AKG (klmno)
v63	PEP (abc) + PEP (def) + E4P (ghij) + Glu (klmno) + ATP + NADPH -> Tyr (abcefghij) + CO_2 (d) + AKG (klmno) + NADH
v64	Ser (abc) + R5P (defgh) + PEP (ijk) + E4P (lmno) + PEP (pqr) + Gln (stuvw) + 3*ATP + NADPH -> Trp (abcedklmnoj) + CO_2 (i) + GAP (fgh) + Pyr (pqr) + Glu (stuvw)
v65	R5P (abcde) + FTHF (f) + Gln (ghijk) + Asp (lmno) + 5*ATP -> His (edcbaf) + AKG (ghijk) + Fum (lmno) + 2*NADH
**One Carbon Metabolism**	
v66	MEETHF (a) + NADH -> METHF (a)
v67	MEETHF (a) -> FTHF (a) + NADPH
